# 4-(4-Chloro­phen­yl)-3-cyano-7-(4-meth­oxy­phen­yl)-5-oxo-5,6,7,8-tetra­hydro-4*H*-chromen-2-aminium methano­late

**DOI:** 10.1107/S1600536812007088

**Published:** 2012-02-24

**Authors:** Rong Sun, Ke Wang, Dong-Dong Wu, Wei Huang, Yang-Bing Ou

**Affiliations:** a Shandong Academy of Chinese Medicine, Jinan 250355, People’s Republic of China; b Postdoctoral Research Station of Shandong University of TCM, Jinan 250355, People’s Republic of China; c Key Laboratory of Nuclear Medicine, Ministry of Health, Jiangsu Key Laboratory of Molecular Nuclear Medicine, Jiangsu Institute of Nuclear Medicine, Wuxi 214063, People’s Republic of China; d Shanghai Institute of Materia Medica, Chinese Academy of Sciences, Shanghai 201203, People’s Republic of China

## Abstract

In the cation of the title organic ion pair compound, C_23_H_20_ClN_2_O_3_^+^·CH_3_O^−^, the cyclo­hexyl ring shows a half-boat conformation and the dihedral angles between two benzene rings and the pyran ring are 83.14 (7) and 73.18 (9)°. In the crystal, centrosymmetrically related cations are linked into a dimer by pairs of N—H⋯N hydrogen bonds, generating an *R*_2_^2^(12) ring motif. The anion inter­acts with the dimer through an N—H⋯O hydrogen bond. π–π inter­actions between pyran rings of adjacent dimers, with a centroid–centroid distance of 3.861 (2) Å, are also observed.

## Related literature

For background to chromene and its derivatives, see: Geen *et al.* (1996 ▶); Ercole *et al.* (2009 ▶); Takakazu *et al.* (2001 ▶). For the synthesis, see: Wen *et al.* (2006 ▶); Kidwai *et al.* (2005 ▶).
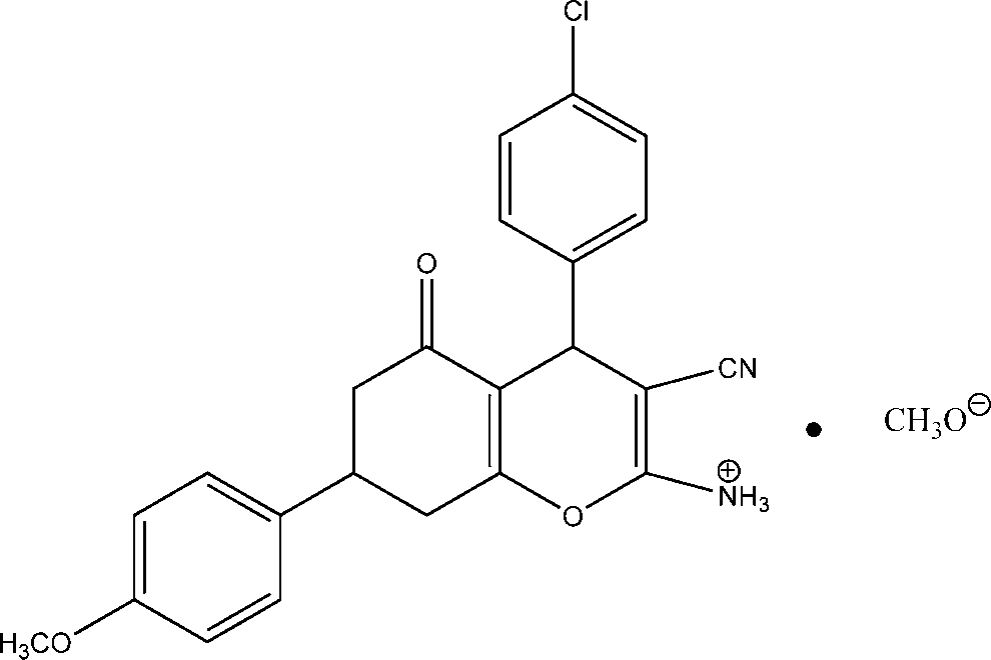


## Experimental

### 

#### Crystal data

C_23_H_20_ClN_2_O_3_^+^·CH_3_O^−^
*M* *_r_* = 438.89
Monoclinic, 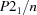

*a* = 8.4408 (12) Å
*b* = 26.844 (4) Å
*c* = 10.4615 (13) Å
β = 100.398 (3)°
*V* = 2331.5 (5) Å^3^
*Z* = 4
Mo *K*α radiation
μ = 0.20 mm^−1^
*T* = 291 K
0.28 × 0.24 × 0.20 mm

#### Data collection

Bruker SMART APEX CCD diffractometer
Absorption correction: multi-scan (*SADABS*; Bruker, 2000 ▶) *T*_min_ = 0.949, *T*_max_ = 0.963
12431 measured reflections
4566 independent reflections
3554 reflections with *I* > 2σ(*I*)
*R*_int_ = 0.042

#### Refinement

*R*[*F* ^2^ > 2σ(*F* ^2^)] = 0.056
*wR*(*F* ^2^) = 0.132
*S* = 1.07
4566 reflections
283 parameters
9 restraints
H-atom parameters constrained
Δρ_max_ = 0.18 e Å^−3^
Δρ_min_ = −0.19 e Å^−3^

### 

Data collection: *SMART* (Bruker, 2000 ▶); cell refinement: *SAINT* (Bruker, 2000 ▶); data reduction: *SAINT*; program(s) used to solve structure: *SHELXS97* (Sheldrick, 2008 ▶); program(s) used to refine structure: *SHELXL97* (Sheldrick, 2008 ▶); molecular graphics: *SHELXTL* (Sheldrick, 2008 ▶); software used to prepare material for publication: *SHELXTL*.

## Supplementary Material

Crystal structure: contains datablock(s) I, global. DOI: 10.1107/S1600536812007088/rz2709sup1.cif

Structure factors: contains datablock(s) I. DOI: 10.1107/S1600536812007088/rz2709Isup2.hkl

Supplementary material file. DOI: 10.1107/S1600536812007088/rz2709Isup3.cml

Additional supplementary materials:  crystallographic information; 3D view; checkCIF report

## Figures and Tables

**Table 1:** Hydrogen-bond geometry (Å, °)

*D*—H⋯*A*	*D*—H	H⋯*A*	*D*⋯*A*	*D*—H⋯*A*
N1—H1*A*⋯O4^i^	0.89	2.12	2.698 (4)	122
N1—H1*B*⋯N2^ii^	0.89	2.26	3.014 (5)	142

Symmetry codes: (i) 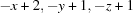; (ii) 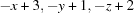.

**Fig. 1.**
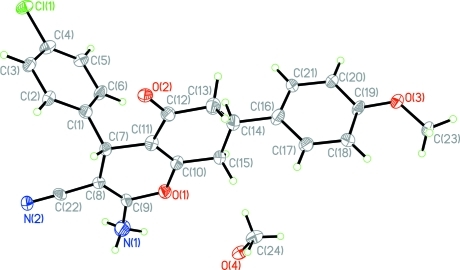
The molecular structure of the title compound, showing 30% probability displacement.

**Fig. 2.**
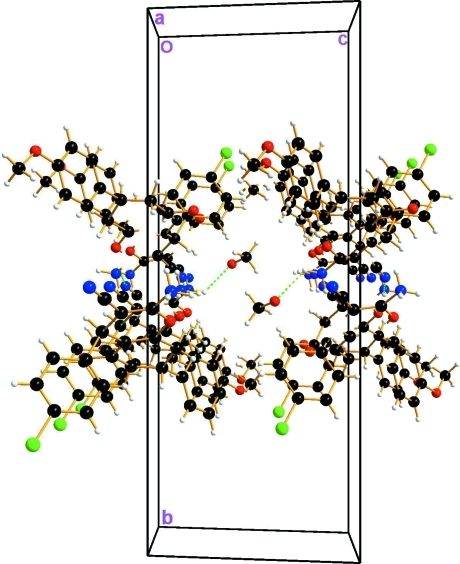
Packing diagram of the title compound viewed along the a axis. Intermolecular hydrogen bonds are shown as dashed lines.

## supplementary crystallographic information

### Comment

Chromenes and their benzo-derivatives are very important heterocyclic compounds, in particular due to their application in a variety of industrial, biological and chemical syntheses (Geen *et al.*, 1996; Ercole *et al.*, 2009; Takakazu *et al.*, 2001). Herein, we report the synthesis and crystal structure of a new chromene derivative.

The molecular structure of the title compound is shown in Figure 1. In the cation of this novel organic ion pair compound, the cyclohexyl ring shows in a half-boat conformation. The dihedral angles between the C1–C6 and C16–C21 benzene rings and the pyran ring are 83.14 (7) and 73.18 (9)°, respectively. In the crystal structure (Fig. 2), centrosymmetrically related cations form a dimer by two intermolecular N—H···N hydrogen bonds (Table 1). Between neighbouring dimers, π–π interactions between pyran rings (centroid-centroid distance = 3.861 (2) Å) are observed. Furthermore, the organic cations and the methanolate anion are linked by intermolecular N—H···O hydrogen bonds.

### Experimental

The title compound were synthesized by the reaction of 4-chlorobenzaldehyde (10 mmol), malononitrile (10 mmol) and 5-(4-methoxyphenyl)-1,3-*cyclo*-hexane-dione (10 mmol) according to the similar synthesis route reported in the literature (Wen *et al.*, 2006; Kidwai *et al.*, 2005). Single crystals of the title compound suitable for X-ray analysis were obtained by slow evaporation of a methanol solution at room temperature for one week.

### Refinement

The aminium H atoms were located in a difference Fourier map ans refined with N—H fixed to 0.89 Å and *U*_iso_(H) = 1.5*U*_eq_(N). All other H atoms were positioned geometrically and refined as riding atoms, with C—H = 0.93–0.98 Å, and with *U*_iso_(H) = 1.2*U*_eq_(C) or 1.5*U*_eq_(N) for methyl H atoms. Rigid bond restraints were applied to the *U*_ij_ values of atoms O1, C4, C5, C10, C14, C15, C17, C18, C20 and C21 with the DELU command in *SHELXL97*.

### Crystal data

**Table:**

C_23_H_20_ClN_2_O_3_^+^·CH_3_O^−^	*F*(000) = 920
*M**_r_* = 438.89	*D*_x_ = 1.250 Mg m^−^^3^
Monoclinic, *P*2_1_/*n*	Mo *K*α radiation, λ = 0.71073 Å
Hall symbol: -P 2yn	Cell parameters from 1476 reflections
*a* = 8.4408 (12) Å	θ = 2.6–19.7°
*b* = 26.844 (4) Å	µ = 0.20 mm^−^^1^
*c* = 10.4615 (13) Å	*T* = 291 K
β = 100.398 (3)°	Block, colourless
*V* = 2331.5 (5) Å^3^	0.28 × 0.24 × 0.20 mm
*Z* = 4	

### Data collection

**Table:**

Bruker SMART APEX CCD diffractometer	4566 independent reflections
Radiation source: sealed tube	3554 reflections with *I* > 2σ(*I*)
Graphite monochromator	*R*_int_ = 0.042
φ and ω scans	θ_max_ = 26.0°, θ_min_ = 2.1°
Absorption correction: multi-scan (*SADABS*; Bruker, 2000)	*h* = −10→10
*T*_min_ = 0.949, *T*_max_ = 0.963	*k* = −26→33
12431 measured reflections	*l* = −8→12

### Refinement

**Table:**

Refinement on *F*^2^	Primary atom site location: structure-invariant direct methods
Least-squares matrix: full	Secondary atom site location: difference Fourier map
*R*[*F*^2^ > 2σ(*F*^2^)] = 0.056	Hydrogen site location: inferred from neighbouring sites
*wR*(*F*^2^) = 0.132	H-atom parameters constrained
*S* = 1.07	*w* = 1/[σ^2^(*F*_o_^2^) + (0.0457*P*)^2^ + 1.6707*P*] where *P* = (*F*_o_^2^ + 2*F*_c_^2^)/3
4566 reflections	(Δ/σ)_max_ = 0.001
283 parameters	Δρ_max_ = 0.18 e Å^−^^3^
9 restraints	Δρ_min_ = −0.19 e Å^−^^3^

### Special details

**Table:**

Experimental. Least-squares planes (*x*,*y*,*z* in crystal coordinates) and deviations from them (* indicates atom used to define plane)7.1912 (0.0049) *x* + 12.2668 (0.0260) *y* - 4.2393 (0.0113) *z* = 8.2055 (0.0187)* -0.0076 (0.0019) C1 * -0.0037 (0.0019) C2 * 0.0134 (0.0020) C3 * -0.0120 (0.0019) C4 * 0.0003 (0.0019) C5 * 0.0095 (0.0020) C6Rms deviation of fitted atoms = 0.0090- 4.4632 (0.0075) *x* + 22.2337 (0.0159) *y* + 2.9074 (0.0097) *z* = 7.7773 (0.0151)Angle to previous plane (with approximate e.s.d.) = 83.14 (7)* -0.0358 (0.0017) C7 * 0.0190 (0.0018) C8 * 0.0065 (0.0017) C9 * -0.0138 (0.0016) O1 * -0.0093 (0.0017) C10 * 0.0334 (0.0018) C11Rms deviation of fitted atoms = 0.0227- 4.8454 (0.0082) *x* - 5.2426 (0.0307) *y* + 9.2662 (0.0059) *z* = 3.3249 (0.0131)Angle to previous plane (with approximate e.s.d.) = 73.18 (9)* -0.0166 (0.0021) C16 * 0.0117 (0.0020) C17 * 0.0054 (0.0019) C18 * -0.0182 (0.0020) C19 * 0.0126 (0.0020) C20 * 0.0050 (0.0021) C21Rms deviation of fitted atoms = 0.0126
Geometry. All e.s.d.'s (except the e.s.d. in the dihedral angle between two l.s. planes) are estimated using the full covariance matrix. The cell e.s.d.'s are taken into account individually in the estimation of e.s.d.'s in distances, angles and torsion angles; correlations between e.s.d.'s in cell parameters are only used when they are defined by crystal symmetry. An approximate (isotropic) treatment of cell e.s.d.'s is used for estimating e.s.d.'s involving l.s. planes.
Refinement. Refinement of *F*^2^ against ALL reflections. The weighted *R*-factor *wR* and goodness of fit *S* are based on *F*^2^, conventional *R*-factors *R* are based on *F*, with *F* set to zero for negative *F*^2^. The threshold expression of *F*^2^ > σ(*F*^2^) is used only for calculating *R*-factors(gt) *etc*. and is not relevant to the choice of reflections for refinement. *R*-factors based on *F*^2^ are statistically about twice as large as those based on *F*, and *R*- factors based on ALL data will be even larger.

### Fractional atomic coordinates and isotropic or equivalent isotropic displacement parameters (Å^2^)

**Table:**

	*x*	*y*	*z*	*U*_iso_*/*U*_eq_	
C1	1.1955 (3)	0.37698 (10)	1.1850 (2)	0.0370 (6)	
C2	1.2679 (3)	0.38035 (10)	1.3165 (3)	0.0414 (6)	
H2	1.2491	0.4078	1.3661	0.050*	
C3	1.3696 (3)	0.34133 (10)	1.3721 (3)	0.0420 (6)	
H3	1.4215	0.3430	1.4584	0.050*	
C4	1.3903 (3)	0.30112 (10)	1.2970 (3)	0.0397 (6)	
C5	1.3237 (3)	0.29740 (10)	1.1703 (3)	0.0404 (6)	
H5	1.3429	0.2697	1.1220	0.048*	
C6	1.2232 (3)	0.33683 (10)	1.1117 (3)	0.0422 (6)	
H6	1.1767	0.3353	1.0242	0.051*	
C7	1.0925 (3)	0.42032 (10)	1.1254 (2)	0.0348 (5)	
H7	1.0677	0.4414	1.1957	0.042*	
C8	1.1785 (3)	0.45122 (9)	1.0401 (2)	0.0335 (5)	
C9	1.1299 (3)	0.45753 (9)	0.9128 (2)	0.0357 (5)	
C10	0.8945 (3)	0.40928 (10)	0.9150 (2)	0.0371 (5)	
C11	0.9333 (3)	0.40222 (10)	1.0433 (2)	0.0349 (5)	
C12	0.8178 (3)	0.37688 (10)	1.1067 (3)	0.0395 (6)	
C13	0.6618 (3)	0.35784 (12)	1.0346 (3)	0.0461 (7)	
H13A	0.6427	0.3248	1.0659	0.055*	
H13B	0.5755	0.3793	1.0512	0.055*	
C14	0.6599 (4)	0.35575 (12)	0.8962 (3)	0.0492 (7)	
H14	0.7259	0.3259	0.8936	0.059*	
C15	0.7507 (3)	0.39052 (10)	0.8273 (2)	0.0384 (5)	
H15A	0.7831	0.3736	0.7543	0.046*	
H15B	0.6821	0.4183	0.7938	0.046*	
C16	0.5059 (3)	0.33767 (11)	0.8126 (3)	0.0454 (7)	
C17	0.3746 (3)	0.37013 (10)	0.7654 (3)	0.0420 (6)	
H17	0.3815	0.4034	0.7903	0.050*	
C18	0.2351 (3)	0.35330 (10)	0.6822 (3)	0.0422 (6)	
H18	0.1502	0.3747	0.6512	0.051*	
C19	0.2300 (3)	0.30415 (9)	0.6492 (3)	0.0382 (6)	
C20	0.3501 (3)	0.27166 (10)	0.6969 (3)	0.0409 (6)	
H20	0.3393	0.2381	0.6751	0.049*	
C21	0.4885 (3)	0.28832 (11)	0.7779 (3)	0.0475 (7)	
H21	0.5704	0.2659	0.8090	0.057*	
C22	1.3256 (3)	0.47351 (9)	1.0965 (2)	0.0336 (5)	
C23	−0.0285 (3)	0.31490 (11)	0.5070 (3)	0.0453 (7)	
H23A	−0.0693	0.3324	0.5742	0.068*	
H23B	−0.1129	0.2951	0.4581	0.068*	
H23C	0.0094	0.3384	0.4503	0.068*	
C24	0.7601 (4)	0.45139 (11)	0.4691 (3)	0.0514 (7)	
H24A	0.6616	0.4430	0.4118	0.077*	
H24B	0.7419	0.4792	0.5224	0.077*	
H24C	0.7964	0.4233	0.5234	0.077*	
Cl1	1.51263 (8)	0.25245 (3)	1.36958 (7)	0.04615 (19)	
N1	1.2003 (3)	0.48469 (10)	0.8308 (3)	0.0576 (7)	
H1A	1.1508	0.4791	0.7496	0.086*	
H1B	1.3035	0.4761	0.8393	0.086*	
H1C	1.1932	0.5169	0.8494	0.086*	
N2	1.4468 (3)	0.49159 (8)	1.1468 (2)	0.0406 (5)	
O1	0.9900 (2)	0.43720 (7)	0.84663 (16)	0.0390 (4)	
O2	0.8398 (2)	0.37310 (7)	1.22654 (17)	0.0427 (5)	
O3	0.1011 (2)	0.28334 (7)	0.56417 (18)	0.0450 (5)	
O4	0.8804 (2)	0.46438 (8)	0.3939 (2)	0.0527 (5)	

### Atomic displacement parameters (Å^2^)

**Table:**

	*U*^11^	*U*^22^	*U*^33^	*U*^12^	*U*^13^	*U*^23^
C1	0.0339 (13)	0.0411 (14)	0.0363 (13)	−0.0051 (11)	0.0073 (11)	0.0039 (11)
C2	0.0393 (14)	0.0445 (15)	0.0361 (14)	0.0022 (12)	−0.0043 (11)	0.0039 (11)
C3	0.0450 (15)	0.0428 (15)	0.0342 (13)	0.0036 (12)	−0.0037 (11)	0.0025 (11)
C4	0.0350 (13)	0.0454 (15)	0.0416 (12)	0.0154 (12)	0.0149 (10)	0.0110 (11)
C5	0.0438 (14)	0.0379 (14)	0.0395 (12)	0.0075 (12)	0.0075 (11)	0.0056 (11)
C6	0.0412 (14)	0.0376 (14)	0.0425 (14)	0.0144 (12)	−0.0063 (11)	−0.0016 (11)
C7	0.0243 (11)	0.0400 (14)	0.0390 (13)	−0.0040 (10)	0.0023 (10)	0.0052 (11)
C8	0.0336 (12)	0.0288 (12)	0.0365 (13)	0.0003 (10)	0.0022 (10)	0.0025 (10)
C9	0.0287 (12)	0.0336 (13)	0.0423 (14)	−0.0044 (10)	−0.0005 (10)	−0.0013 (11)
C10	0.0327 (12)	0.0427 (14)	0.0348 (13)	0.0024 (10)	0.0032 (9)	0.0024 (10)
C11	0.0294 (12)	0.0444 (14)	0.0324 (12)	0.0032 (11)	0.0091 (10)	−0.0027 (11)
C12	0.0320 (13)	0.0447 (15)	0.0429 (15)	−0.0038 (11)	0.0095 (11)	0.0070 (12)
C13	0.0394 (15)	0.0538 (17)	0.0440 (15)	−0.0005 (13)	0.0042 (12)	0.0026 (13)
C14	0.0443 (15)	0.0549 (17)	0.0443 (16)	−0.0075 (12)	−0.0028 (12)	0.0041 (13)
C15	0.0368 (12)	0.0387 (14)	0.0363 (13)	−0.0032 (10)	−0.0020 (10)	0.0004 (10)
C16	0.0443 (15)	0.0448 (16)	0.0416 (15)	0.0130 (12)	−0.0069 (12)	−0.0059 (12)
C17	0.0387 (13)	0.0426 (15)	0.0444 (15)	0.0020 (11)	0.0067 (11)	0.0074 (12)
C18	0.0410 (14)	0.0388 (14)	0.0446 (15)	−0.0056 (11)	0.0023 (11)	0.0068 (11)
C19	0.0335 (13)	0.0352 (13)	0.0418 (14)	0.0139 (11)	−0.0038 (11)	−0.0037 (11)
C20	0.0331 (13)	0.0365 (14)	0.0500 (15)	0.0051 (11)	−0.0009 (11)	0.0061 (11)
C21	0.0398 (14)	0.0450 (16)	0.0517 (16)	−0.0135 (12)	−0.0074 (12)	0.0141 (13)
C22	0.0317 (13)	0.0297 (12)	0.0410 (13)	−0.0070 (10)	0.0106 (11)	0.0027 (10)
C23	0.0370 (14)	0.0477 (16)	0.0441 (15)	0.0007 (12)	−0.0122 (11)	0.0103 (12)
C24	0.0565 (18)	0.0480 (17)	0.0529 (17)	0.0150 (14)	0.0186 (14)	0.0118 (14)
Cl1	0.0419 (4)	0.0492 (4)	0.0497 (4)	0.0160 (3)	0.0148 (3)	0.0153 (3)
N1	0.0484 (14)	0.0671 (17)	0.0570 (15)	−0.0033 (13)	0.0080 (12)	0.0131 (13)
N2	0.0376 (12)	0.0419 (12)	0.0391 (12)	−0.0072 (10)	−0.0020 (9)	0.0002 (10)
O1	0.0356 (9)	0.0435 (10)	0.0372 (10)	−0.0063 (8)	0.0046 (7)	0.0024 (8)
O2	0.0417 (10)	0.0461 (11)	0.0390 (10)	−0.0109 (8)	0.0037 (8)	0.0156 (8)
O3	0.0397 (10)	0.0438 (11)	0.0458 (11)	0.0018 (8)	−0.0075 (8)	0.0069 (8)
O4	0.0561 (12)	0.0491 (12)	0.0532 (12)	0.0147 (10)	0.0108 (10)	0.0063 (9)

### Geometric parameters (Å, º)

**Table:**

C1—C6	1.367 (4)	C14—C15	1.476 (4)
C1—C2	1.404 (4)	C14—C16	1.509 (4)
C1—C7	1.518 (4)	C14—H14	0.9800
C2—C3	1.411 (4)	C15—H15A	0.9700
C2—H2	0.9300	C15—H15B	0.9700
C3—C4	1.365 (4)	C16—C21	1.375 (4)
C3—H3	0.9300	C16—C17	1.426 (4)
C4—C5	1.347 (4)	C17—C18	1.407 (4)
C4—Cl1	1.751 (3)	C17—H17	0.9300
C5—C6	1.424 (3)	C18—C19	1.363 (4)
C5—H5	0.9300	C18—H18	0.9300
C6—H6	0.9300	C19—C20	1.362 (3)
C7—C8	1.499 (3)	C19—O3	1.392 (3)
C7—C11	1.537 (3)	C20—C21	1.388 (4)
C7—H7	0.9800	C20—H20	0.9300
C8—C9	1.331 (3)	C21—H21	0.9300
C8—C22	1.408 (3)	C22—N2	1.168 (3)
C9—N1	1.343 (3)	C23—O3	1.427 (3)
C9—O1	1.370 (3)	C23—H23A	0.9600
C10—C11	1.336 (3)	C23—H23B	0.9600
C10—O1	1.390 (3)	C23—H23C	0.9600
C10—C15	1.472 (3)	C24—O4	1.435 (3)
C11—C12	1.445 (3)	C24—H24A	0.9600
C12—O2	1.238 (3)	C24—H24B	0.9600
C12—C13	1.485 (4)	C24—H24C	0.9600
C13—C14	1.446 (4)	N1—H1A	0.8900
C13—H13A	0.9700	N1—H1B	0.8900
C13—H13B	0.9700	N1—H1C	0.8900
			
C6—C1—C2	120.8 (2)	C13—C14—H14	98.9
C6—C1—C7	121.2 (2)	C15—C14—H14	98.9
C2—C1—C7	118.0 (2)	C16—C14—H14	98.9
C1—C2—C3	118.7 (3)	C10—C15—C14	110.8 (2)
C1—C2—H2	120.6	C10—C15—H15A	109.5
C3—C2—H2	120.6	C14—C15—H15A	109.5
C4—C3—C2	118.8 (2)	C10—C15—H15B	109.5
C4—C3—H3	120.6	C14—C15—H15B	109.5
C2—C3—H3	120.6	H15A—C15—H15B	108.1
C5—C4—C3	123.4 (2)	C21—C16—C17	117.3 (3)
C5—C4—Cl1	118.7 (2)	C21—C16—C14	120.2 (3)
C3—C4—Cl1	117.8 (2)	C17—C16—C14	122.6 (3)
C4—C5—C6	118.6 (3)	C18—C17—C16	122.0 (3)
C4—C5—H5	120.7	C18—C17—H17	119.0
C6—C5—H5	120.7	C16—C17—H17	119.0
C1—C6—C5	119.6 (2)	C19—C18—C17	116.9 (3)
C1—C6—H6	120.2	C19—C18—H18	121.6
C5—C6—H6	120.2	C17—C18—H18	121.6
C8—C7—C1	111.4 (2)	C20—C19—C18	122.7 (3)
C8—C7—C11	108.1 (2)	C20—C19—O3	114.9 (2)
C1—C7—C11	111.4 (2)	C18—C19—O3	122.4 (2)
C8—C7—H7	108.6	C19—C20—C21	120.4 (3)
C1—C7—H7	108.6	C19—C20—H20	119.8
C11—C7—H7	108.6	C21—C20—H20	119.8
C9—C8—C22	117.0 (2)	C16—C21—C20	120.7 (3)
C9—C8—C7	124.8 (2)	C16—C21—H21	119.7
C22—C8—C7	118.1 (2)	C20—C21—H21	119.7
C8—C9—N1	128.0 (2)	N2—C22—C8	178.0 (3)
C8—C9—O1	122.5 (2)	O3—C23—H23A	109.5
N1—C9—O1	109.5 (2)	O3—C23—H23B	109.5
C11—C10—O1	122.2 (2)	H23A—C23—H23B	109.5
C11—C10—C15	127.1 (2)	O3—C23—H23C	109.5
O1—C10—C15	110.7 (2)	H23A—C23—H23C	109.5
C10—C11—C12	117.7 (2)	H23B—C23—H23C	109.5
C10—C11—C7	123.2 (2)	O4—C24—H24A	109.5
C12—C11—C7	119.1 (2)	O4—C24—H24B	109.5
O2—C12—C11	120.9 (2)	H24A—C24—H24B	109.5
O2—C12—C13	116.3 (2)	O4—C24—H24C	109.5
C11—C12—C13	122.6 (2)	H24A—C24—H24C	109.5
C14—C13—C12	111.8 (2)	H24B—C24—H24C	109.5
C14—C13—H13A	109.3	C9—N1—H1A	109.5
C12—C13—H13A	109.3	C9—N1—H1B	109.5
C14—C13—H13B	109.3	H1A—N1—H1B	109.5
C12—C13—H13B	109.3	C9—N1—H1C	109.5
H13A—C13—H13B	107.9	H1A—N1—H1C	109.5
C13—C14—C15	123.5 (3)	H1B—N1—H1C	109.5
C13—C14—C16	116.5 (3)	C9—O1—C10	118.80 (19)
C15—C14—C16	112.9 (2)	C19—O3—C23	118.8 (2)
			
C6—C1—C2—C3	−0.2 (4)	C7—C11—C12—O2	5.7 (4)
C7—C1—C2—C3	−177.4 (2)	C10—C11—C12—C13	1.3 (4)
C1—C2—C3—C4	−1.8 (4)	C7—C11—C12—C13	−179.5 (2)
C2—C3—C4—C5	2.7 (4)	O2—C12—C13—C14	−169.7 (3)
C2—C3—C4—Cl1	−178.2 (2)	C11—C12—C13—C14	15.3 (4)
C3—C4—C5—C6	−1.5 (4)	C12—C13—C14—C15	−30.8 (4)
Cl1—C4—C5—C6	179.5 (2)	C12—C13—C14—C16	−179.1 (2)
C2—C1—C6—C5	1.4 (4)	C11—C10—C15—C14	−7.9 (4)
C7—C1—C6—C5	178.5 (2)	O1—C10—C15—C14	172.8 (2)
C4—C5—C6—C1	−0.6 (4)	C13—C14—C15—C10	27.5 (4)
C6—C1—C7—C8	−72.7 (3)	C16—C14—C15—C10	176.7 (2)
C2—C1—C7—C8	104.4 (3)	C13—C14—C16—C21	−94.4 (4)
C6—C1—C7—C11	48.2 (3)	C15—C14—C16—C21	114.1 (3)
C2—C1—C7—C11	−134.7 (2)	C13—C14—C16—C17	86.3 (4)
C1—C7—C8—C9	117.3 (3)	C15—C14—C16—C17	−65.2 (4)
C11—C7—C8—C9	−5.5 (3)	C21—C16—C17—C18	−2.6 (4)
C1—C7—C8—C22	−59.6 (3)	C14—C16—C17—C18	176.7 (3)
C11—C7—C8—C22	177.6 (2)	C16—C17—C18—C19	0.5 (4)
C22—C8—C9—N1	−3.3 (4)	C17—C18—C19—C20	2.4 (4)
C7—C8—C9—N1	179.7 (3)	C17—C18—C19—O3	−177.8 (2)
C22—C8—C9—O1	179.2 (2)	C18—C19—C20—C21	−3.1 (4)
C7—C8—C9—O1	2.3 (4)	O3—C19—C20—C21	177.0 (3)
O1—C10—C11—C12	173.9 (2)	C17—C16—C21—C20	1.9 (4)
C15—C10—C11—C12	−5.3 (4)	C14—C16—C21—C20	−177.4 (3)
O1—C10—C11—C7	−5.2 (4)	C19—C20—C21—C16	0.8 (4)
C15—C10—C11—C7	175.6 (2)	C8—C9—O1—C10	0.6 (4)
C8—C7—C11—C10	6.9 (3)	N1—C9—O1—C10	−177.3 (2)
C1—C7—C11—C10	−115.9 (3)	C11—C10—O1—C9	1.0 (4)
C8—C7—C11—C12	−172.2 (2)	C15—C10—O1—C9	−179.7 (2)
C1—C7—C11—C12	65.0 (3)	C20—C19—O3—C23	−178.5 (2)
C10—C11—C12—O2	−173.5 (3)	C18—C19—O3—C23	1.6 (4)

### Hydrogen-bond geometry (Å, º)

**Table:**

*D*—H···*A*	*D*—H	H···*A*	*D*···*A*	*D*—H···*A*
N1—H1*A*···O4^i^	0.89	2.12	2.698 (4)	122
N1—H1*B*···N2^ii^	0.89	2.26	3.014 (5)	142

Symmetry codes: (i) −*x*+2, −*y*+1, −*z*+1; (ii) −*x*+3, −*y*+1, −*z*+2.
